# A Xeno-Free Strategy for Derivation of Human Umbilical Vein Endothelial Cells and Wharton's Jelly Derived Mesenchymal Stromal Cells: A Feasibility Study toward Personal Cell and Vascular Based Therapy

**DOI:** 10.1155/2020/8832052

**Published:** 2020-09-07

**Authors:** Hataiwan Kunkanjanawan, Tanut Kunkanjanawan, Veerapol Khemarangsan, Rungrueang Yodsheewan, Kasem Theerakittayakorn, Rangsun Parnpai

**Affiliations:** ^1^Medeze Research and Development Co., Ltd, 28/9 Moo 8, Phutthamonthon Sai 4 Rd., Krathum Lom, Sam Phran, Nakhon Pathom 73220, Thailand; ^2^Medeze Stem Cell Co., Ltd, 28/9 Moo 8, Phutthamonthon Sai 4 Rd., Krathum Lom, Sam Phran, Nakhon Pathom 73220, Thailand; ^3^Department of Pathology, Faculty of Veterinary Medicine, Kasetsart University, 1 Moo 6, Kamphaeng Saen, Nakhon Pathom 73140, Thailand; ^4^Embryo Technology and Stem Cell Research Center, School of Biotechnology, Suranaree University of Technology, 111 University Avenue, Muang Nakhon Ratchasima, Nakhon Ratchasima 30000, Thailand

## Abstract

Coimplantation of endothelial cells (ECs) and mesenchymal stromal cells (MSCs) into the transplantation site could be a feasible option to achieve a sufficient level of graft-host vascularization. To find a suitable source of tissue that provides a large number of high-quality ECs and MSCs suited for future clinical application, we developed a simplified xeno-free strategy for isolation of human umbilical vein endothelial cells (HUVECs) and Wharton's jelly-derived mesenchymal stromal cells (WJ-MSCs) from the same umbilical cord. We also assessed whether the coculture of HUVECs and WJ-MSCs derived from the same umbilical cord (autogenic cell source) or from different umbilical cords (allogenic cell sources) had an impact on *in vitro* angiogenic capacity. We found that HUVECs grown in 5 ng/ml epidermal growth factor (EGF) supplemented xeno-free condition showed higher proliferation potential compared to other conditions. HUVECs and WJ-MSCs obtained from this technic show an endothelial lineage (CD31 and von Willebrand factor) and MSC (CD73, CD90, and CD105) immunophenotype characteristic with high purity, respectively. It was also found that only the coculture of HUVEC/WJ-MSC, but not HUVEC or WJ-MSC mono-culture, provides a positive effect on vessel-like structure (VLS) formation, *in vitro*. Further investigations are needed to clarify the pros and cons of using autogenic or allogenic source of EC/MSC in tissue engineering applications. To the best of our knowledge, this study offers a simple, but reliable, xeno-free strategy to establish ECs and MSCs from the same umbilical cord, a new opportunity to facilitate the development of personal cell-based therapy.

## 1. Introduction

Since blood supply is an essential factor that holds the great effect on graft survival and host tissue integration, various approaches promoting vascularization have been developed in the field of tissue engineering research. Among these, cotransplantation of multiple cell types (i.e., adipose-derived stem cells and human umbilical vein endothelial cells; HUVECs) has proven to yield a superiority effect on neovascularization in an adipogenesis mouse models [1]. This finding is in accordance with Ma et al. (2014), who showed that coculture of human adipose tissue-derived or human bone marrow-derived mesenchymal stromal cells (MSCs) with HUVECs resulted in vessel-like structure (VLS) formation after *in vivo* implantation, either on day 3 or on day 7, in athymic mouse models [2]. However, although the beneficial effects between MSCs and ECs have been reported [3–5], these studies were performed on MSCs and ECs derived from the different individuals, as an allogenic cell source. Little is known about the angiogenic capacity of MSCs and ECs coculturing especially when those cells derived from the same (autogenic) source.

Human umbilical cord (hUC) is a unique niche that contains abundant source of postnatal stem cells (such as haematopoietic stem cells and MSCs) and ECs (such as HUVECs) [3, 4, 6]. Several groups have reported various protocols for the isolation of Wharton's jelly-derived mesenchymal stromal cells (WJ-MSCs) from hUC using animal-free or so-called xeno-free culture system [7–10]. Xeno-free culture system refers to the cell cultivation processes that avoid the use of animal-associated supplement, such as fetal bovine serum (FBS) and porcine trypsin, due to an awareness on contamination; both from xenogenic compound and microorganism. Nowadays, xeno-free culture strategy includes, but not limit to, the use of human blood derivatives (such as human serum and human platelet lysate), microbial recombinant proteins, and chemically defined media [11]. Indeed, the advantage of xeno-free culture system is not only to eliminate the risk of zoonosis but also to promote self-renewal ability and multilineage differentiation potential [7, 12, 13]. Over the past few decades, numerous studies illustrate the great value of MSCs in the field of tissue engineering and regenerative medicine through their differentiation potential, ability to homing and engraftment, and paracrine factors secretion [14]. However, one of the major obstacles to transfer this upcoming technology to clinical use is the culture system that the cells have been established. Therefore, to comply with the long-term safety requirements for cell-based therapy, xeno-free established cells have become a preferred source of cell-based product suited for future clinical application [15].

To creating a new opportunity to facilitate the development of personal cell and vascular-based therapy, the objectives of this study are to isolate and expand HUVECs and WJ-MSCs from the same umbilical cord using the defined xeno-free strategies and to determine how the coculture of autogenic and allogenic HUVEC/WJ-MSC contribute to the angiogenic capacity, *in vitro*.

## 2. Materials and Methods

### 2.1. Chemicals and Media

All reagents were purchased from Sigma-Aldrich (St. Louis, MO, USA) unless otherwise stated.

### 2.2. Isolation of HUVECs and WJ-MSCs

In this study, ethical approval was granted by the Ethics Committee for Researches Involving Human Subjects, Suranaree University of Technology (EC-62-81), Nakhon Ratchasima, Thailand. After receiving the signed informed consents from the parents, the umbilical cords (*N* = 3) were collected and processed at Medeze stem cell laboratory within 24 hrs after delivery. In all experiments, cells were maintained in a humidified atmosphere of 37^๐^C and 5% CO_2_ incubator.

HUVECs were isolated from umbilical vein as described previously [16], with some modification. Briefly, the collected umbilical cords were sterilized by ethanol and rinsed twice by phosphate-buffered saline (PBS). Then, the umbilical vein was filled with 0.2% collagenase (xeno-free grade, EMD Milipore; Cat. No. SCR139) and incubated at room temperature for 30 min. After that, the cells were collected and cultured on 25 cm^2^ tissue culture flask (Corning). Three different media were examined for their effects on HUVECs isolation: (a) commercial xeno-culture (nonxeno-free) system composed of basal medium 200 (Invitrogen) supplemented with low serum growth supplements (LSGS kit, contain 2% v/v FBS, Invitrogen); (b) xeno-free culture system composed of M199/EBSS (Hyclone) containing 10% human serum (HS), 2 mM L-glutamine, 10 ng/ml basic fibroblast growth factor (bFGF, Prospec), 5 U/ml heparin (xeno-free grade, Life science production), 100 U/ml penicillin, and 100 *μ*g/ml streptomycin (Millipore); (c) xeno-free culture system (B) supplemented with 5 ng/ml epidermal growth factor (EGF, Prospec). These 3 conditions were next referred as LSGS, xeno-free+bFGF, and xeno-free+bFGF+EGF, respectively. On the following day, the media were changed to remove cell debris. The culture media were refreshed every 3-4 days.

After HUVECs isolation, the same umbilical cord was subjected to isolate WJ-MSCs by using tissue explant method. In brief, the umbilical vein was mechanically excised and removed. Then, the umbilical cord matrix was cut into 2-3 mm thick and cultured on 20 *μ*g/ml human fibronectin coated tissue culture plate. WJ-MSCs were maintained in *α*MEM (Hyclone) supplemented with 10% HS, 100 U/ml penicillin, and 100 *μ*g/ml streptomycin. The culture media were refreshed every 3-4 days.

At 90% confluence, the HUVECs and WJ-MSCs were subcultured by TrypLE Express (Invitrogen) and used for subsequent studies. For future use, HUVECs and WJ-MSCs were frozen in freezing medium consisting of culture medium plus 10% dimethylsulfoxide (Wak-Chemie Medical GmbH) and 20% (*v*/*v*) human serum albumin solution (Baxter). The frozen aliquots were then stored in vapor phase liquid nitrogen.

### 2.3. Immunocytochemical Staining

The expression of key endothelial markers, including CD31 (PECAM-1) and von Willebrand factor (vWf), was used to qualitatively analyzed HUVECs cell lines (*N* = 3) obtained from each condition. At passage 3, HUVECs were seeded onto 4-well tissue culture plate (Nunc) and fixed with 4% paraformaldehyde after reaching confluence. Then, cells were washed and blocked with 10% fetal bovine serum in 0.25% Triton X-100 for 1 hr. The cells were incubated with the primary antibodies against human CD31 (1 : 200, Abcam) and human vWf (1 : 200, Abcam) overnight at 4^๐^C. Subsequently, cells were washed and incubated for 1 hr with appropriate Alexa 488 (Invitrogen)/DyLight 594 (Thermo Fisher Scientific) conjugated secondary antibody. After washing, cell nuclei were stained with 4′,6-diamidino-2-phenylindole (DAPI, Biolegend) for 10 min at room temperature. Finally, the cells were observed under an inverted fluorescent microscope.

### 2.4. Expansion Potential of HUVECs under Different Culture Conditions

To compare expansion potential in each culture condition, growth kinetics of HUVECs (from passage 4 to passage 6) obtained from LSGS, xeno-free+bFGF, and xeno-free+bFGF+EGF conditions were quantified as described previously [17], with some modifications. Briefly, HUVECs (*N* = 3) were plated at 10,000 cells/cm^2^ and cultured under their originated condition for 72 hrs. Then, cells from each group were collected, stained with 0.4% trypan blue (Invitrogen), and counted by Countess™ Automated Cell Counter (Invitrogen). Each condition was performed in duplicate. Population doubling (PD) was calculated using equation (1). NH is the harvested cell number, and NI is the initial cell number.

(1) $$\begin{array}{c} \text{PD}=\frac{\log_{10}\text{NH}-\log_{10}\text{NI}}{\log_{10}2}. \end{array}$$

### 2.5. Flow Cytometry Analysis

The purities of HUVECs (at passage 3 and passage 6, each passage *N* = 3) and WJ-MSCs (at passage 3, *N* = 3) were taken for flow cytometry analysis. The following antihuman antibodies were used according to the manufacturer's instructions. For HUVECs, cells were stained with anti-CD31-FITC, anti-CD105-PE, and anti-CD45-FITC. For WJ-MSCs, cells were stained with anti-CD73-APC, anti-CD90-FITC, anti-CD105-PE, and anti-CD45-FITC. All antibodies were purchased from BD Bioscience. Corresponding isotype immunoglobulins were used as negative control. Single-cell suspensions of 1 × 10^5^ cells were incubated with the appropriate concentration antibodies for 20 min at room temperature. Then, cells were washed and resuspended in 500 *μ*L PBS and analyzed using CytoFLEX flow cytometer (Beckman Coulter). At least 10,000 events were acquired, and the results were analyzed using CytExpert software.

### 2.6. *In vitro* 2D-Angiogenic Capacity and Immunocytochemical Staining

An *in vitro* angiogenic capacities of monocultures and cocultures of autogenic and allogenic HUVECs/WJ-MSCs (1 : 1 cell ratio) were determined by 2-dimensional (2D) culture (*N* = 3), as previously described [2] with some modifications. In the autogenic group, HUVECs and WJ-MSCs derived from the single umbilical cord were cocultured together. In the allogenic group, HUVECs and WJ-MSCs derived from the different umbilical cords were cocultured together. A total number of 2 × 10^5^ cells were seeded and cultured in basal medium 200 supplemented with LSGS on 0.2% gelatin-coated 4-well tissue culture plates. At day 3, samples were washed by PBS and fixed with 4% paraformaldehyde for 20 minutes.

For immunocytochemical staining, 5% normal goat serum was used as a blocking solution for 1 hr at room temperature. After that, samples were incubated with rabbit antihuman CD31 polyclonal antibody (1 : 80, Abcam) at 4^๐^C, overnight. After three times washing by 0.05% PBS-Tween, the goat anti-rabbit conjugated-horseradish peroxidase (1 : 1000, Abcam) was added into the cultured wells for 1 hr at room temperature. After washing, 3,3′-Diaminobenzidine (DAB) was used as substrate, and hematoxylin was next applied for nuclear staining. The stained cells were observed, and the photos were taken by using an inverted microscope.

### 2.7. Statistical Analysis

All experiments were performed on 3 different cell lines with duplicate. Data were expressed as mean ± SEM. Statistical analysis was performed by SPSS software using one-way ANOVA and paired, two-tailed Student's *t*-test. A *P* value was considered statistically significant different at *P* < 0.05.

## 3. Results

### 3.1. Xeno-Free System Support the Isolation and Expansion of HUVECs and WJ-MSCs

**Figure 1:**
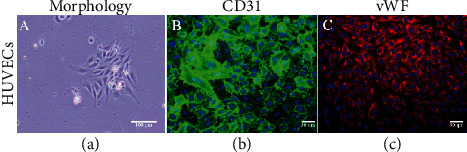
Growth colony and the expression of endothelial cell markers of isolated HUVECs. A typical cobblestone-like colony was observed after 24 hours culture (a). A representative image of (b) CD31 (green) and (c) vWF (red) was detected in HUVECs regardless of their cultivated condition. Nuclei of the cells were indicated by 4′,6-diamidino-2-phenylindole staining. Studies were performed on three independent cell lines with duplicates. Scale bar: 100 *μ*m (a) and 50 *μ*m (b, c).

To obtain multiple cell types potentially useful for cell and vascular based therapy, we first examined the possibility of isolation of HUVECs and WJ-MSCs from single umbilical cord using a well-defined xeno-free culture system compared with commercially available xeno-containing culture medium. In this study, we were able to obtain both HUVEC and WJ-MSC cell lines from all donors under tested culture conditions. Based on phase-contrast microscopic appearance, HUVECs from all three conditions demonstrated a classical cobblestone-like morphology (Figure 1(a)) with no significant difference observed. After reaching confluence of primary culture, HUVECs from all three conditions were able to be passaged, expanded in monolayer culture, and cryopreserved for future usage. At passage 3, the expressions of endothelial-specific markers were first confirmed by indirect immunofluorescence staining. We found that the expressions of CD31 and vWF were detected in HUVECs obtained from all three conditions (Figures 1(b) and 1(c)).

After 7-14 days of culture, adherent fibroblast-like cell populations were grown out from the edge of WJ explants. These cells also had the ability to expand and can be cryopreserved for future study. Therefore, by using a simple tissue explant technic, the rest of the umbilical cord can still be used as a source of WJ-MSCs.

### 3.2. Xeno-Free Culture System Accelerated the Proliferation Potential of HUVECs

**Figure 2:**
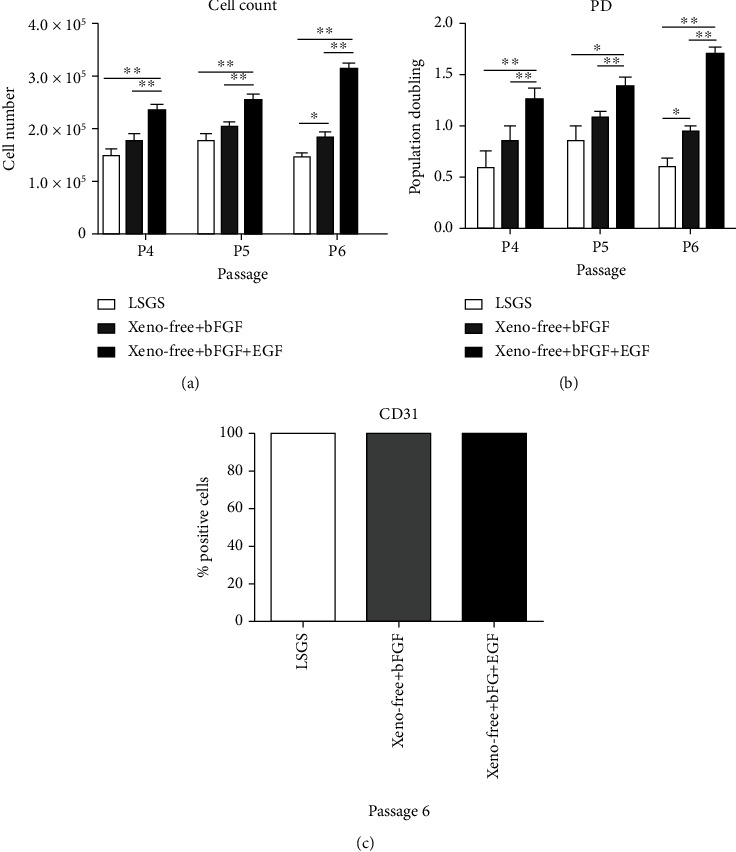
Effects of different culture constituents on growth kinetics and endothelial marker maintenance of HUVECs. Comparison of LSGS, xeno-free+bFGF, and xeno-free+bFGF+EGF culture media on cell proliferation (a) and population doubling (b) at passage 4, 5, and 6. At passage 6, HUVECs obtained from all three conditions still maintain the expression of CD31 (c). Data were expressed as mean ± SEM (*N* = 3). Significant differences at ^∗^*P* < 0.05 and ^∗∗^*P* < 0.01.

We next evaluated the effect of media constituent and supplement on the proliferation of HUVECs isolated from all three conditions: LSGS, xeno-free+bFGF, and xeno-free+bFGF+EGF. We found that HUVECs cultured in xeno-free+bFGF+EGF condition exhibited a higher proliferation activity than the other media tested (*P* < 0.01; Figure 2(a)). As shown by population doubling plots (P4 and P6 (*P* < 0.01) and P5 (*P* < 0.05); Figure 2(b)), the accelerated expansions of HUVECs under xeno-free+bFGF+EGF condition were observed at all-time point assessed. Moreover, it was noteworthy that the expression of CD31 remained stable for HUVECs obtained from all three conditions after 6 passages (Figure 2(c)). Based on these experimental findings, HUVECs isolated under xeno-free+bFGF+EGF condition were then selected for the subsequent experiment.

### 3.3. Expanded HUVECs and WJ-MSCs Showed Their Immunophenotype Characteristics with High Purity

**Figure 3:**
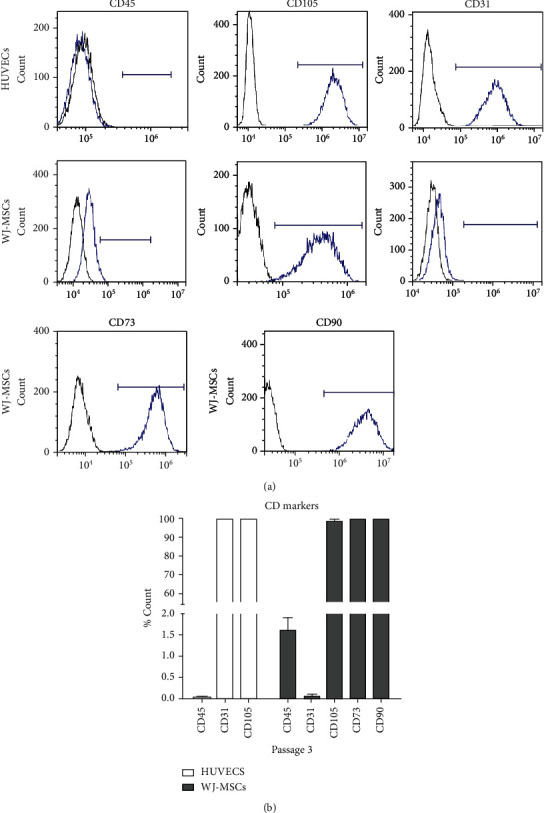
Purification of HUVECs and WJ-MSCs obtained by using a single umbilical cord with xeno-free cell isolation and expansion strategy. (a) Representative flow cytometry analysis of expanded HUVECs and WJ-MSCs stained with antibodies directed against human antigens specific for pan leukocyte (CD45), ECs (CD31), and MSCs: CD105, CD73, and CD90. (b) Percentage of HUVECs (light bar) and WJ-MSC-s (gray bar) stained positive for endothelial and MSC markers. Data were expressed as mean ± SEM (*N* = 3).

Since cell purity is one of the key parameters required for cellular therapy products, we next performed flow cytometry analysis for both HUVECs and WJ-MSCs obtained by our technics. At passage 3, the percentage of CD31^+^ (99.96% ± 0.05%), CD105^+^ (99.70% ± 0.13%), and CD45^−^ (0.01% ± 0.02%) cells indicated that HUVECs isolated under xeno-free+bFGF+EGF condition possess endothelial lineage characteristics [18] with more than 99% purity (Figures 3(a) and 3(b)). And, WJ-MSCs isolated from the remaining umbilical cord were fulfill the classical MSC immunophenotype [19]; cells were positive for CD73 (99.72% ± 0.15%), CD90 (99.75% ± 0.21%), and CD105 (98.83% ± 0.65%) and were negative for CD45 (1.61% ± 0.54%) (Figures 3(a) and 3(b)).

### 3.4. *In vitro* 2-Dimensional Coculture of HUVECs/WJ-MSCs Exhibited a Vessel-Like Structure

**Figure 4:**
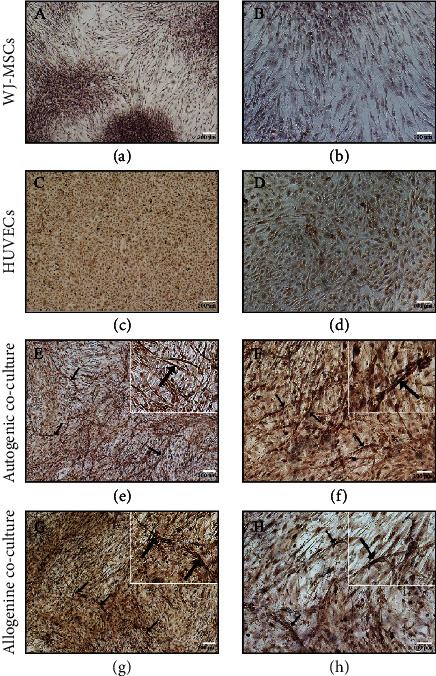
Vessel-like structure formation in 2-dimensional (2D) culture. After 3 days cultured in endothelial growth media, WJ-MSCs (a, b), HUVECs (c, d), autogenic (e, f), and allogenic (g, h) HUVECs/WJ-MSCs coculture were stained with antibodies directed against human CD31 (brown). Arrows indicated CD31 stained VLS in both of autogenic and allogenic HUVECs/WJ-MSCs coculture condition. Scale bar: 200 *μ*m (a, c, e, and g) and 100 *μ*m (b, d, f, and h).

To assess whether cocultured of ECs and WJ-MSCs has positive effects on angiogenicity, HUVECs obtained from xeno-free+bFGF+EGF condition were then cocultured with WJ-MSCs for 3 days. Immunostaining of CD31 was used to confirm the endothelial lineage phenotype of the structure. We found that the vessel-like structures (VLS) stained positively for CD31 were observed only in HUVECs/WJ-MSCs coculture in both of autologous (Figures 4(e) and 4(f)) and allogenic (Figures 4(g) and 4(h)) coculture conditions. In contrast, none of VLS was identified in HUVECs (Figures 4(c) and 4(d)) and WJ-MSCs (Figures 4(a) and 4(b)) monoculture.

## 4. Discussion

To achieve a desirable therapeutic outcome in the field of stem cells and tissue engineering, multidisciplinary factors should be taken into consideration, for example, a performance of engineered tissues, an appropriate structural/scaffold support, and a sufficient oxygen and nutrient supply of engineered tissue. [20]. To overcome the problem of inadequate blood supply, several strategies that promote graft-host vascularization have been employed: (i) direct derivation of proangiogenic growth factors such as vascular endothelial growth factor, fibroblast growth factor, and platelet-derived growth factors [21]; (ii) the transplantation of multiple cells types (i.e., endothelial progenitor cells; EPCs, ECs, and MSCs) [2, 5, 22, 23]; and (iii) the use of vascular-inductive biocompatible scaffolds combined with stem/progenitor cells [24]. ECs can be isolated from different types of blood vessels, such as arteries, veins, and capillaries [25]. For instance, autologous EC sources are internal mammary artery, saphenous vein, and skin tissue [26, 27]. However, harvesting of autologous ECs is usually not possible because of the invasive collection procedure, poor tissue quality cause by preexisting conditions of the patient, and limited proliferation potential of the isolated cells. hUC, as HUVEC origin, in contrast, offers a high-quality EC source that is readily available after a child's birth. The advantages of HUVECs beyond other EC source include sample accessibility, uncomplicate isolation process, and availability of published data [27].

In the present study, the attention has been drawn to the use of cells obtained from hUC, a potential cell source that is routinely discarded after birth. Apart from its ethical acceptance and painless collection procedure, hUC provides the unique niche of multiple types of cell (i.e., WJ-MSCs [4], ECs, and EPCs from umbilical cord vein [28, 29]) that provide a mutual support mechanism through their inheritance distinct functions, multilineage differentiation potentials, and neovascularization [30, 31]. Here, we report for the first time that HUVECs and WJ-MSCs can be obtained from the same umbilical cord using a simple 2-step technic, the first enzymatic digestion of the umbilical vein followed by mechanical explant of Wharton's jelly tissue. This technic utilized the fact that hUC contains different types of multipotent progenitor cells, and these cells itself have a natural barrier to prevent cell line cross-contamination during the isolation process. We also demonstrated that medium comprised of 10% human serum, an appropriate xeno-free alternative of FBS, could be used to support the expansion, until cryopreservation, of HUVECs and WJ-MSCs. To get the superior expansion condition for HUVECs, we found that the addition of 5 ng/ml EGF yielded a better proliferation potential when compared with the standard xeno-free+bFGF culture condition and nonxeno-free LSGS culture condition, respectively. The results of flow cytometry analysis showed that both HUVECs and WJ-MSCs obtained by this technic possess the immunophenotype characteristic of endothelial and MSCs with more than 99% and 98% purity, respectively. This is in accordance with the previous report that hUC can be used as a source for vascular cell bank [16]. It is worth mentioning that, besides the establishment of xeno-free culture system for both WJ-MSCs and HUVECs, our findings fulfill the attempt to establish therapeutic applicable cell bank [32–36] by offering a possibility to set up “two-in-one” (two cell types in one sample) autogenic cell bank.

The ability of ECs and MSCs on neovascularization is also a topic of interest in the field of stem cells and tissue engineering. A number of studies have proven that different sources of MSCs (e.g., bone marrow-derived and adipose tissue-derived MSCs) provide beneficial effects on vascular tube formation of ECs via stabilization of ECs network and secretion of vasculogenic growth factor, such as hepatocyte growth factor [37, 38]. However, the promising results of those studies obtained from the coculturing of allogenic cell sources, which might be less attractive considering in personalized cell therapy [39]. In this study, we found that only coculture of HUVECs/WJ-MSCs was able to form VLS after 3 days culture in endothelial culture medium. These implied the angiogenic supporting effect of HUVECs and WJ-MSCs in both of autogenic and allogenic cell source conditions. Although, we were not able to perform the quantitative comparison between VLS degree derived from autogenic and allogenic cell source conditions. Our preliminary results provide compelling evidence that the coculturing of the autogenic and allogenic HUVECs/WJ-MSCs give rise to the different level of VLS formation. Further investigations on molecular interactions, a quantitative assay of VLS formation, and an *in vivo* functional study of these cells are needed to clarify which combination (autogenic or allogenic cell source) should be considered before moving from the bench to the bedside.

In conclusion, the defined xeno-free strategy proposed by this study offers a simple, but reliable, approach to establish the autogenic HUVECs and WJ-MSCs that are suited for future personal clinical application.

## Data Availability

Source data used to support the findings of this study are available from the corresponding author upon request.
